# Statistical Measures to Quantify Similarity between Molecular Dynamics Simulation Trajectories

**DOI:** 10.3390/e19120646

**Published:** 2017-11-29

**Authors:** Jenny Farmer, Fareeha Kanwal, Nikita Nikulsin, Matthew C. B. Tsilimigras, Donald J. Jacobs

**Affiliations:** 1 Department of Bioinformatics and Genomics, University of North Carolina at Charlotte, Charlotte, NC 28223, USA; 2 Department of Physics and Optical Science, University of North Carolina at Charlotte, Charlotte, NC 28223, USA; 3 Center for Biomedical Engineering and Science, University of North Carolina at Charlotte, Charlotte, NC 28223, USA

**Keywords:** molecular dynamics, conformational fluctuations, conformational similarity measures, *p*-values, statistical significance, beta-lactamase, site directed mutations

## Abstract

Molecular dynamics simulation is commonly employed to explore protein dynamics. Despite the disparate timescales between functional mechanisms and molecular dynamics (MD) trajectories, functional differences are often inferred from differences in conformational ensembles between two proteins in structure-function studies that investigate the effect of mutations. A common measure to quantify differences in dynamics is the root mean square fluctuation (RMSF) about the average position of residues defined by C*α*-atoms. Using six MD trajectories describing three native/mutant pairs of beta-lactamase, we make comparisons with additional measures that include Jensen-Shannon, modifications of Kullback-Leibler divergence, and local *p*-values from 1-sample Kolmogorov-Smirnov tests. These additional measures require knowing a probability density function, which we estimate by using a nonparametric maximum entropy method that quantifies rare events well. The same measures are applied to distance fluctuations between C*α*-atom pairs. Results from several implementations for quantitative comparison of a pair of MD trajectories are made based on fluctuations for on-residue and residue-residue local dynamics. We conclude that there is almost always a statistically significant difference between pairs of 100 ns all-atom simulations on moderate-sized proteins as evident from extraordinarily low *p*-values.

## 1. Introduction

Since the snapshots of three-dimensional structures of proteins were first captured with X-ray crystallography, many tens of thousands of protein structures have been resolved, allowing structural biologists to infer the remarkable connection between molecular shape and biological function [1,2]. Although the large accumulation of protein structure snapshots is invaluable in the foundation of structural biology, there are inherent limitations to extracting information from this data. Proteins are not static structures, but rather highly flexible, adaptable, and dynamic macromolecules where conformational change oftentimes plays a key role in a mechanistic process governing protein function. Understanding conformational dynamics is critical to predicting how proteins function [3–6].

Molecular dynamics (MD) simulation is a very powerful approach that allows the motions of a protein to be investigated in exquisite detail. MD explores molecular dynamics through a series of time frames by solving dynamical equations of motions based on models that capture the essential features of molecular and atomic interactions [7,8]. In principle, MD is a brute force method that samples the response of a protein under a particular set of conditions, which includes random motions from thermal fluctuations. As such, a MD simulation trajectory represents a sample, or a single instance, of what a protein could do in its dynamics. Given many potential bifurcation points along a trajectory, the number of ways a protein can respond to a given condition is astronomical. Nevertheless, proteins are observed to function with high fidelity in reproducibility, suggesting the existence of certain pathways in conformational dynamics that are frequently visited. Therefore, a pertinent question is how much information about functional mechanisms can be extracted from a statistical sample obtained from MD simulation? Consider, for example, that an all-atom MD simulation requires a time-step of about 1 femtosecond to numerically solve a complicated system of coupled differential equations [9,10]. Extrapolating upward from this base timescale, a sense of how effective a MD simulation can sample functional mechanisms can be obtained by reviewing the typical timescales important for function as determined from experiment.

Usually spanning more than 12 orders of magnitude in timescales, ranging from picoseconds to seconds, protein dynamics captures conformational fluctuations occurring on small length scales to large structural rearrangements that occur in folding and unfolding events [3,4,11–14]. Therefore, an ensemble of conformations represents a protein better than one static structure [15]. Biologically important timescales are typically in the range of microseconds to seconds [16–19]. To sample this range of timescales using MD is therefore generally unattainable. Fortunately, exploring the entire ensemble of conformations is unnecessary in practice, bypassed by analyzing conditional ensembles that describe a particular aspect of protein dynamics, such as within the native state. However, conditional ensembles must connect, making simulation of protein dynamics challenging. Pursuit of overcoming these challenges has led to myriad forms of MD simulation methods to generate functionally relevant conformational ensembles. An increasing trend in structural bioinformatics is to facilitate comparative analysis for the similarity and differences between conformational ensembles [20–26]. A nagging question about whether such comparisons are meaningful deals with whether the MD simulation has converged [27–34].

While it appears to us that overall robustness of MD simulations is not statistically reliable in regard to conformational sampling, it is undeniable that MD offers a powerful tool to glean insight into molecular mechanisms. As a tool, albeit imperfect, the use of MD has ubiquitously permeated into the field of biochemistry, and has substantially advanced our understanding of how proteins function. With continual increases in computing power, improved algorithms and parameterization for MD simulations, both the accuracy of the results and the biological timescales that can be reached has increased steadily. Insights into mechanisms governing molecular binding that are afforded by MD simulation also show promise for computer-aided drug design. The dichotomy between sampling incompleteness and practical utility prompted us to benchmark conformational similarity between a pair of MD trajectories using a variety of statistical measures. It has become commonplace to simulate protein motions in the native state using all-atom MD simulations in explicit solvent to compare a pair of similar proteins, such as wild type versus mutant. Subsequent analysis of the MD trajectories looks for significant differences and similarities to infer mechanisms responsible for function, if any. We aim to check if differences/similarities can be quantitatively discerned between two proteins using relatively short MD trajectories, of say 50 to 100 ns.

Structure-function studies are routinely performed in biochemistry to understand the effects of mutations and elucidate mechanisms. Mutations in proteins spontaneously occur in nature as well. With pressure on living organisms to survive when subjected to stress, a protein sequence can evolve to prevent extinction. An important biomedical example impacting human health is how mutations alter the function of *β*-lactamase [35], a bacterial enzyme that causes the majority of antibiotic resistance [36,37]. In particular, TEM-1 *β*-lactamase is capable of hydrolyzing penicillin (meaning it confers resistance to it) and first generation cephalosporins, but it does not confer resistant to extended-spectrum antibiotics (i.e., oxyimino cephalosporins) [38]. TEM-1 is the longest known and is among the most commonly found *β*-lactamase within bacteria and hence is also referred to as the parental TEM. As antibiotics are administered to kill bacteria, mutated *β*-lactamases confer resistance to an extended spectrum of antibiotics that can differ from TEM-1 by just a few substitution mutations. Here, we consider 3-point and 1-point mutations as examples. The MD trajectories of TEM-1 and its mutants should be distinguishable with statistical significance if their known functional differences are to be discerned using this method of simulation.

We limit the scope of this work to address differences in dynamics in terms of fluctuations about the average position of residues based on C*α* atoms, and distances between pairs of C*α* atoms within some cutoff distance. In this paper, we present results pertaining to a 12 Å cutoff distance, and results for smaller cutoff distances are provided in supporting information. Our expectation is that the dominant collective modes defined by principal component analysis (PCA) [39] should, by their very nature, lead to results with equal or greater levels of similarity than results based on local dynamical variables. However, to our knowledge, residue-level comparisons have not been done before to the extent presented here. In particular, we employ new methodology that leverages an accurate nonparametric probability distribution function estimation [40,41] to capture rare events. In short, we revisit previously identified troubling issues, but quantify similarity of local dynamics more precisely to determine the scale of statistical significance (or lack thereof) between trajectory comparisons. As a control, we study the self-consistency between the first and second half of all simulation trajectories. Based on three pairs of proteins comparing TEM-1 *β*-lactamase with a mutant, we consider a set of statistical measures while exploring a few alternative implementations. Questions about the influence of starting structures, convergence, and differences in levels of statistical significance between different MD trajectories for the same wildtype protein compared to its mutated structures are addressed. Interestingly, we find there is no similarity in samples involving local variables in the usual sense of *p*-values greater than 0.05 under all circumstances. Rather, extraordinarily small *p*-values form the basis for a comparative metric for relative statistical significance to quantify sampling differences.

## 2. Materials and Methods

### Dataset

We consider three *β*-lactamase X-ray crystal structures 1erm, 1htz, 1li9 from the Protein Data Bank [42–44]. All of the structures have been solved to high resolution (average = 1.8 Å) and have R-values ≤ 0.23. 1htz is a hexamer in the P 4_3_ 2_1_ 2 space group and the other two structures are monomers in the P 2_1_ 2_1_ 2_1_ space group. We use the TEM-1 sequence (263 residues) as a standard reference. Point mutations are introduced using the rotamer explore functionality within the Molecular Operating Environment (MOE) software. 1erm had at position 189 a (3*S*)-3-hydroxy-L-aspartic acid that was converted to aspartic acid. Hydrogen atoms and structural minimization are performed with GROMACS 4.6.1, with the AMBER99-SB-ILDN force field and TIP3P explicit water. All structures are protonated with GROMACS-preferred protonation of histidine residues in 1erm. The disulfide bond between the cysteine pair is enabled.

The 1li9 and 1htz structures correspond to extended spectrum TEM-34 and TEM-52 *β*-lactamases (also having 263 residues), and they perfectly align with TEM-1, referenced as wildtype (WT). The single point mutation MET044VAL transforms TEM-1 to TEM-34. Three point mutations: GLU079LYS, MET157THR, GLY213SER, transform TEM-1 to TEM-52. Again using MOE to make substitution mutants, we computationally reverse mutated TEM-34 and TEM-52 back to TEM-1, as well as mutated the TEM-1 sequence using the 1erm structure to TEM-52. This process provides three pairs of WT/mutant structure comparisons, where two of the mutants are the same in terms of sequence, but based on different X-ray crystal starting structures. When preparing the monomer structures for an all-atom MD simulation in explicit water, we select the first chain available whenever the X-ray crystal structure has multiple chains, and, select the first alternate conformation whenever there are alternate conformations. The *β*-lactamase simulations were chosen partially because of their biological importance, and because we expect the results obtained on statistical significance and utility of various statistical metrics will generalize to all MD simulations.

### Molecular Dynamics Simulations

We performed six production-run MD simulations using the GPU-accelerated GROMACS 4.6.1 [45,46] using the same force field mentioned previously. Structures were solvated using TIP3P water [47], using comparable numbers of water molecules and box sizes for all simulations. A minimum of 1 nm buffer was used between the protein and side of a box. Sodium ions were added to neutralize the system. Steepest descent minimization with a tolerance of 1000 kJ/(mol·m) was performed until convergence. The system was subsequently equilibrated using position-restrained 1 ns of NVT followed by 1 ns of NPT, as verified by inspection of equilibration for temperature (for NVT) and density (for NPT). The modified Nosé-Hoover thermostat [48] was used to control temperature. The Parrinello-Rahman barostat [48] was used to control pressure at 1 atm. The Verlet cutoff scheme was used [49], which is a requirement of GPU-accelerated GROMACS. Particle mesh Ewald was used for long-range electrostatics, with a cutoff of 1 nm. LINCS was used to constrain bonds to hydrogen atoms [50], and SETTLE was applied to water molecules [51], which together allows for a simulation time-step of 2 fs. Production runs in the NVT ensemble are 100. Coordinates were saved every 50 ps, yielding 2000 frames per MD trajectory. This protocol has been established in prior work, and we have found it to be a good set of common parameters that works well against diverse proteins across a range of sizes.

### Nonparametric density estimation

The most common form for probability density estimation is based on kernel density estimation (KDE). In our experience, we find the results of KDE to be too dependent on human subjectivity because the default bandwidth frequently does not yield a correct probability density function (PDF) [52]. Note that KDE frequently requires improvements to be sought by having an “expert user” adjust the bandwidth. More problematic, the convolution of the true PDF with the kernel function (usually a Gaussian distribution) causes systematic errors in the tails of the PDF, which is a critical part of a PDF, reflecting boundary conditions. If rare events are of interest, an automated high throughput alternative to KDE that is not subjective is required. Therefore we employ an in-house nonparametric density estimation method previously developed to address these issues [40,41], which has some similarity with maximum likelihood methods.

Calculus of variation is used to determine an appropriate form for a PDF that maximizes entropy, subject to a variety of constraints using the method of Lagrange multipliers. The form of the equation for the PDF is as follows

(1) $$P(v)=\exp\left(\lambda_{0}-1+\sum_{j=1}^{D}\lambda_{j}g_{j}(v)\right)$$

where {*λ_j_*} are a set of *D* Lagrange multipliers and {*g_j_*(*v*)} are a set of complete orthogonal level functions, chosen in this work as Chebyshev polynomials [52]. The Lagrange multipliers, and the dimension *D*, are determined computationally through an iterative random search method. Trial Lagrange multipliers are used to calculate a PDF, which is then evaluated according to how well it matches the random data sample. To perform this evaluation, the cumulative distribution function is calculated from the trial PDF, and is used to map the sampled data to a random sample on the interval [0, 1]. For the true PDF, the mapped sample data will represent sampled uniform random data (SURD) on the interval [0, 1]. Statistical characteristics of SURD were quantified through single order statistics. Probability functions for single order statistics are known, being beta distributions, and calculating them is straightforward [53]. A likelihood function is constructed as a product of the individual probability functions. The exact characteristics of this constructed likelihood function is described by a universal sample size invariant scoring function. This means, an exact confidence level can be determined for any trial PDF as to whether it is the true PDF, without knowing what the true PDF is. Consequently it is possible to generate an ensemble of good PDF estimates.

The method we employ selects the most representative PDF among a five-member ensemble of good PDF estimates. Accuracy in the selected PDF estimate is commensurate with the number of samples, and the final results are highly resistant to over- or under-fitting with no need for a user to know about any property of the distribution. Although not presented here, a large number of tests were made to quantify the sensitivity of the results for this application. Since the results are very robust, we present no error analysis on the PDF estimates. Details about the nonparametric density estimation can be found in an online archive [40], and Java code is available upon request.

### Statistical measures

Consider two different random samples labeled as *a* and *b* each having *N* random variables given as {*x_k_*}*_a_* and {*y_k_*}*_b_* where *k* runs from 1 to *N*. For each sample the standard deviation is empirically calculated, denoted as *RMSF*(*a*) and *RMSF*(*b*). The estimated PDF for sample *a* is denoted as *p*(*x*|*a*), and likewise *p*(*y*|*b*) refers to sample *b*. For sample *a*, the corresponding cumulative distribution function (CDF) is denoted as *F*(*x*|*a*), and likewise *F*(*y*|*b*) refers to sample *b*. For reference, we note that the Kullback-Leibler divergence [54] is defined as

(2) $$KL[q(z),p(z)]=\int ln\;\left[\frac{q(z)}{p(z)}\right]\;q(z)\;dz$$

where *z* is a continuous random variable, *q*(*z*) is the true population probability density, and *p*(*z*) is an estimated probability density from a sample. Although the form of *KL* suggests it can provide useful information about the similarity between the statistics of two samples, it cannot be applied in its original form because the true distribution is unknown in this work. Following prior work in the context of quantifying similarity between conformational ensembles [21], this problem has been resolved by using the Jensen-Shannon measure (*JS*) that symmetrizes the *KL* measure while replacing the true PDF with the average PDF estimates, such that $q(z)\to \frac{1}{2}[p(z|a)+p(z|b)]$.

We calculate eight non-negative statistical measures for similarity between samples *a* and *b*, defined as:

(3) $$\Delta \mathrm{RMSF}(a,b)=\;|{\mathrm{RMSF}}_{a}-{\mathrm{RMSF}}_{b}|$$

(4) $$JS(a,b)=\frac{KL[q(z),p(z|a)]+KL[q(z),p(z|b)]}{2}$$

(5) Δp(a,b)=∫∣p(z∣a)-p(z∣b)∣dz

(6) $$\mathrm{KLmin}(a,b)=\int \left|ln\;\left[\frac{p(z|a)}{p(z|b)}\right]\right|\min[p(z|a),p(z|b)]\;dz$$

(7) $$\mathrm{KLave}(a,b)=\int \left|ln\;\left[\frac{p(z|a)}{p(z|b)}\right]\right|\sqrt{p(z|a)p(z|b)}\;dz$$

(8) $$\mathrm{KLmax}(a,b)=\int \left|ln\;\left[\frac{p(z|a)}{p(z|b)}\right]\right|\max[p(z|a),p(z|b)]\;dz$$

(9) $$KS1(a,b)=\sqrt{KS1(a|b)KS1(b|a)}$$

(10) $$KS0(a,b)=\sup_{x}|F(z|a)-F(z|b)|$$

In (9) $KS1(a|b)=\sup_{x}\left|F_{n}^{\left(a\right)}-F(z|b)\right|$ is the Kolmogorov-Smirnov 1-sample test [55] where *F_n_*^(^*^a^*^)^ is the empirical cumulative distribution based on observations from sample *a*, and *F*(*z*|*b*) is the CDF estimated from sample *b*. Likewise, $KS1(b|a)=\sup_{x}\left|F_{n}^{\left(b\right)}-F(z|a)\right|$. Therefore, it is seen that *KS*1(*a*, *b*) is symmetrized by taking a geometric average of *KS*1(*a*|*b*) and *KS*1(*b*|*a*). We created in (10) a “0-sample” test that uses the two estimated CDFs. We note that standard two-sample tests were compared with the 1 and 0 sample tests, and, with the exception of more noise, the results for *p*-values were in agreement across all methods. This consistency serves as another indicator that statistical resolution and systematic errors in the PDF estimates have no noticeable effect in this application. We use kstest() MATLAB-2017a function to convert KS measures to *p*-values.

Our motivation for introducing the three measures, *KLmin*, *KLave*, and *KLmax* is that the argument |ln *p*(*z*|*a*)/*p*(*z*|*b*)| is a non-negative measure for the deviation between the two probability distributions in the same spirit as the KL divergence. However, not knowing the true PDF, it is represented by the lowest estimate in (6), the highest estimate in (8) and the geometrical average in (7). These measures are not normalized in the sense that ∫ max[*p*(*z*|*a*), *p*(*z*, *b*)] *dz* ≤ 1, ∫ min[*p*(*z*|*a*), *p*(*z*, *b*)] *dz* < 1, and $\int \sqrt{p(z|a)p(z,b)}dz<1$. The last inequality holds because a geometric mean is always equal to or less than an arithmetic mean. As such, *KSave* serves the same purpose as *JS*, while *KLave* has definitive bounds given as *KLmin* ≤ *KLave* ≤ *KLmax*. It is worth mentioning that the measure *KLmin* will suppress the effect of outliers while *KLmax* will emphasize outliers, and thus the occurrence of rare events.

### Data extraction

For all results presented here, the MD trajectories had an initial frame0 that was the last frame from the equilibration process. At 50 ps intervals, 2000 frames were generated. The first and second sets of 1000 frames define samples 1 and 2. If convergence is monitored in protein *A*, the two samples compared are *A*_1_ and *A*_2_. If traits in proteins *A* and *B* are compared, four pairs of samples are tested as: {*A*_1_&*B*_1_, *A*_2_&*B*_1_, *A*_1_&*B*_2_, *A*_2_&*B*_2_}. Hence, the sample size is 1000 observations in all cases. When determining the magnitude of displacements from the average position of a C*α* atom, it is noted that GROMACS is used to align all frames of the trajectory to a reference structure. We define self-alignment to indicate frame0 of a MD trajectory is used to align all other frames to it so that global translations and rotations are removed before taking average positions of C*α* atoms. We define mean-alignment to indicate an average structure is used to align all MD runs, where the average structure reflects only the six different frame0 structures (3 WT and 3 mutants), and not the average over all frames and all MD trajectories. Note that an average over the entire set of MD frames and trajectories was deemed unnecessary (results shown below).

Throughout our analysis we employ local averaging within samples. Given samples 1 and 2, their respective empirical averages *μ*_1_ and *μ*_2_ define the local-averages. Statistical fluctuations generally cause these averages to be unequal even if the samples are drawn from the same PDF. In contrast, a global-average assigns the same average to both samples, given as *μ* = (*μ*_1_ + *μ*_2_)/2 since both samples are the same size. This distinction may be relevant for random variables representing displacements from an average position because differences in the first moment of corresponding distributions is likely to decrease similarity measures. We address a similar concern regarding distance pairs using an alternate procedure called shifted distribution. In this case, a local-average is assigned to samples, and, after the PDF is estimated, the calculated mean of that PDF is used in a linear transformation to shift the random variables as well as the PDF. This shifted distribution characterizes fluctuations about a mean, and all means after the shift are precisely zero. With only displacement information retained, similarity measures can only improve, or they will remain the same if the local-averages of both samples happened to be identical.

### Data collapse

We reduce the amount of data to compare by averaging data in various ways. In some cases, the averaging procedure improves the robustness of the statistical measure, otherwise it is done for convenience. After calculating statistical measures *M_kj_*(*a*, *b*) associated with residues *k* and *j* involving two different sample sets, we collapse the dataset of all residue pairs {*M_kj_*(*a*, *b*)} onto an average *M̄_k_*(*a*, *b*) for the k-th residue. This average is an arithmetic mean over all nearest neighbors of residue *k*. Two residues are considered nearest neighbors if their C*α* atom distances are less than or equal to 12 Å for at least one frame in the entire MD trajectory. If samples *a* and *b* are drawn from two different MD trajectories, the corresponding residue pairs must be nearest neighbors within each sample, otherwise the comparison is not made. In other words, when comparing two different trajectories, we take the intersection of residue pairs. Since we are comparing sequences with perfect alignment (point mutation differences only) this intersection method always works. This local spatial averaging reduces fluctuations in *M̄_k_*(*a*, *b*) relative to the original residue pairs, and improves robustness in comparisons along the backbone of the proteins.

Averaging pairwise comparisons between two half time-intervals also increases robustness in the statistical measures. We analyze MD trajectories in two halves so that a single MD trajectory has two equal samples. When two different proteins are compared, this gives a total of four possible comparisons (e.g., {*A*_1_&*B*_1_, *A*_2_&*B*_1_, *A*_1_&*B*_2_, *A*_2_&*B*_2_}). We perform an arithmetic mean over these four cases. For example, for pairwise quantities, we arrive at a single average measure given as $\langle {\bar{M}}_{k}(A,B)\rangle =\frac{1}{4}[{\bar{M}}_{k}(A_{1},B_{1})+{\bar{M}}_{k}(A_{2},B_{1})+{\bar{M}}_{k}(A_{1},B_{2})+{\bar{M}}_{k}(A_{2},B_{2})]$. Averaging over 4 time-blocks is also applied to on-residue quantities.

## 3. Results

### 3.1. Molecular Dynamics Trajectory Comparisons

We analyze data across a variety of groups as summarized in Table 1. For convenience the structure/sequence cases we consider are referenced in Table 1 as A = 1erm:wt, B = 1htz:wt, C = 1li9:wt, X = 1erm:m3, Y = 1htz:m3, Z = 1li9:m1, where wt is the wildtype, m3 is the 3-point mutation, m1 is the 1-point mutation, and mt simply denotes a mutation. The respective correspondence between A, B, C to X, Y, Z indicates that the same structure from the protein data bank is used as a starting point, where mutation differences between the two proteins is performed computationally as explained in the methods and materials section. We use 1R to denote on-residue measures involving C*α* atom displacements from an average position, which requires alignment. The per residue measure obtained by averaging all nearest neighbor distance pairs as explained in the methods and materials section is denoted by 2R. We note that essentially no difference between measures based on global and local averages was observed when testing convergence in dataset groups 1R-self-1 and 1R-self-2. Since local averaging is easier to work with computationally, we do not present separate results based on global averaging.

### 3.2. Identifying Similar Similarity Measures

**Figure 1:**
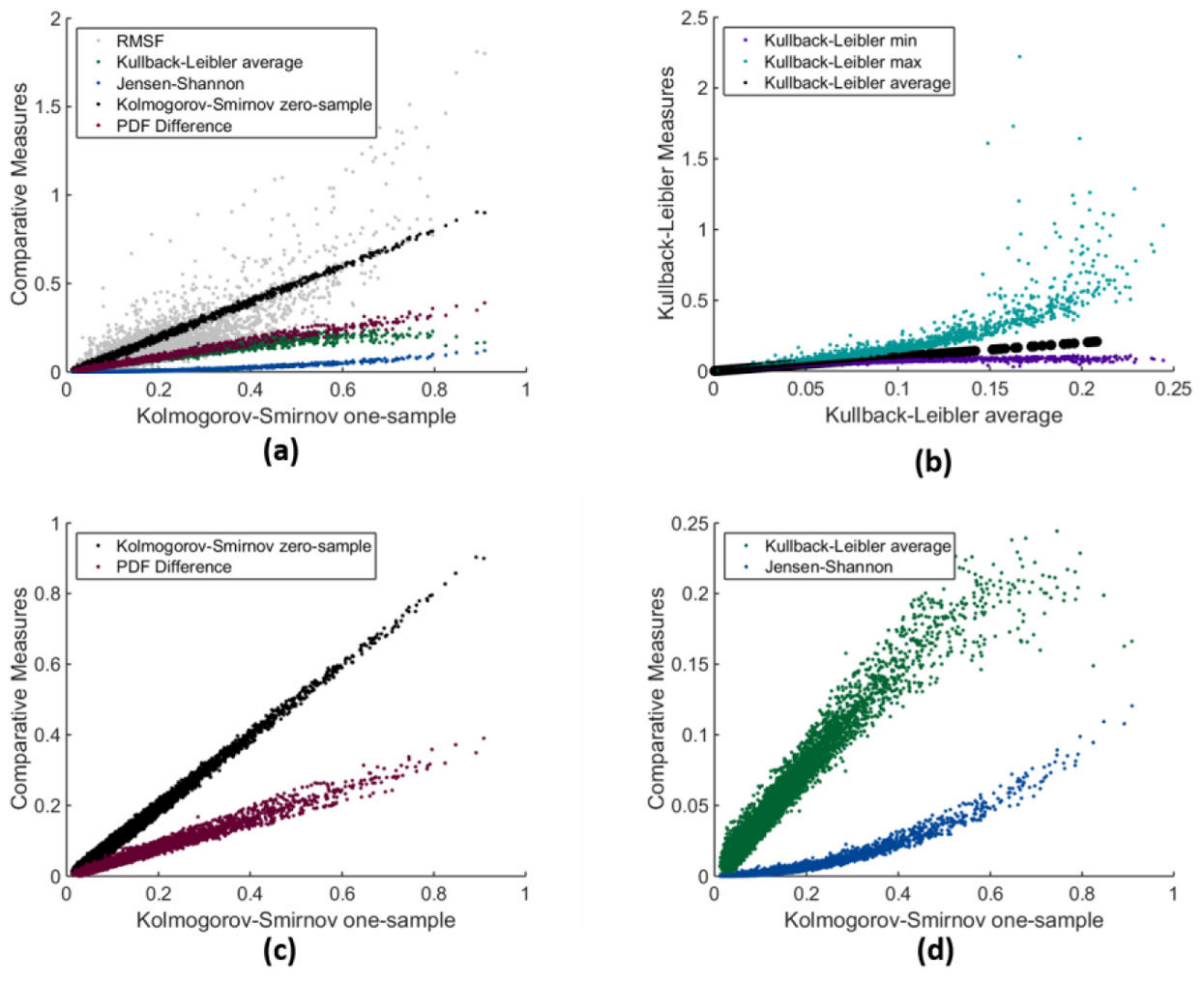
Comparison of various measures: (**a**) All statistical measures, except for *KS*1, *KLmin* and *KLmax*, are plotted on a scatter plot against the *KS*1 measure; (**b**) As a scatter plot *KLmin* and *KLmax* are plotted against *KLave*; (**c**) Δ*p* and *KS*0 are plotted on a scatter plot against the *KS*1 measure; (**d**) *JS* and *KLave* are plotted on a scatter plot against the *KS*1 measure.

A common approach for comparing two MD trajectories is to plot RMSF from each trajectory against each other, and interpret large differences as a potential indicator for differences in function. Among all the measures we consider, we first address which set of measures are similar to RMSF and which measures provide some orthogonal information. In Figure 1, typical scatter plots are shown with various measures on the *y*-axis and *KS*1 on the *x*-axis. The scatter plots include all residue to corresponding residue comparisons that are contained within the groups 1R-wt-wt-1, 1R-wt-wt-2, 1R-mt-wt-1 and 1R-mt-wt-2. Among the many combinations of possible pairs explored, Figure 1 exemplifies our findings. First, RMSF is the least correlated to all other measures. Second, many pairs of measures are effectively redundant, tracking each other through a linear relationship well. Third, *RMSF*, *KS*1, *KLave* and *JS* contain the greatest amount of orthogonal information. Although *KLmax* tracks *KLave* fairly well, Figure 1b shows that for large *KLave*, *KLmax* is often much larger than proportional due to a non-linear upward curvature in the scatter. This is not surprising because *KLmax* accentuates the effect of outliers, which is where *KLave* is large.

### 3.3. Comparison of Alignment Methods

**Figure 2:**
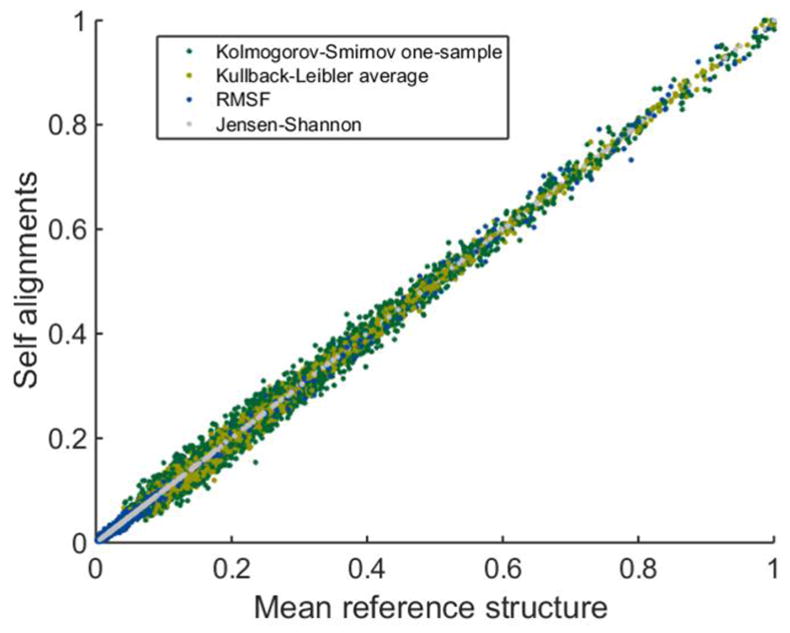
Comparison of four on-residue measures using two different reference structures.

Based on groups 1R-wt-wt-1, 1R-wt-wt-2, 1R-mt-wt-1 and 1R-mt-wt-2, we investigated if the reference structure used in the alignment of trajectory frames impacts the on-residue measures. It is clear from Figure 2 that the results obtained using the mean reference structure are practically the same as that when using self-alignments per MD trajectory across the four most distinct measures. A third possible alignment strategy would be to find the average structure across the entire ensemble of frames for all simulations, but considering how little difference was found between the mean-alignment and self-alignment methods, this third alignment method was not pursued. Instead, the choice of reference structure is bypassed completely by measuring distance pairs, discussed in the next section.

### 3.4. Improving Dynamical Information by Removing Structural Effects

Statistical information about distance pairs is rotationally invariant, meaning the results are independent of a reference structure. Hence, structural alignment of the MD frames is not necessary for 2R-descriptions. Nevertheless, the PDF obtained using local averaging retains information about the structure because the mean value of a distance pair defines an equilibrium length when protein dynamics has quasi-converged. Even if the simulation is not converged, from a statistical sample, each distance pair will have an average value that is empirically calculated. These “equilibrium lengths” reflect the average structure of the protein over the simulation time. It is possible that the average structure can mask the local dynamics of the protein, which can be better described by displacements about the equilibrium length of a particular distance pair. To represent distance fluctuations without regard to the underlying protein structure, we shift the variables so that the first moment of a distance pair PDF is redefined to be zero (see methods and materials).

**Figure 3:**
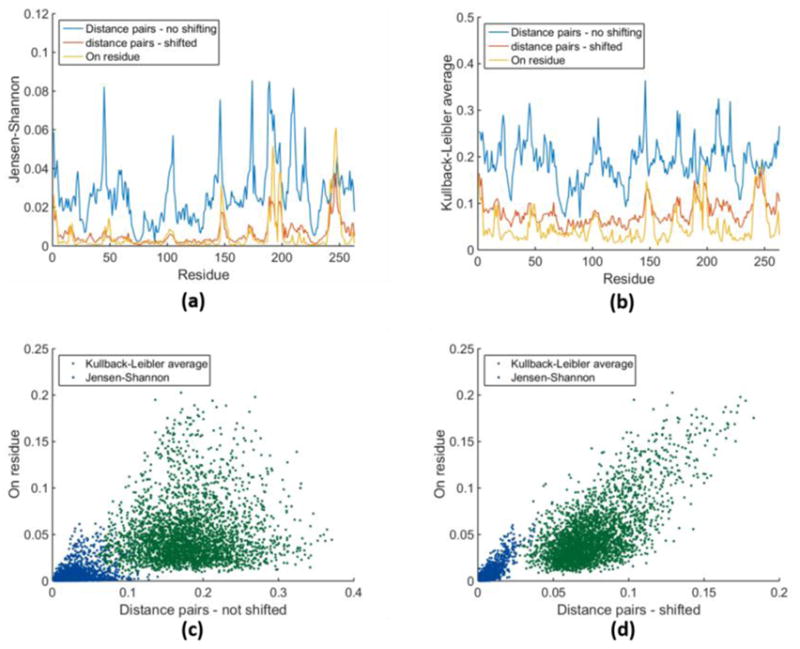
Comparison of distance pair description with on-residue description. (**a**) As a typical case, the *JS* measure calculated in three different ways is plotted along the backbone of the protein for a wt to wt comparison using the 1erm and 1li9 structures. (**b**) Same as (**a**) but using the *KLave* measure. (**c**) Scatter plot of the R2 description on the *y*-axis against the R1 description for both the *JS* and *KLave* measures. (**d**) Same as (**c**) but the measures are shifted.

The on-residue descriptions from dataset groups (1R-wt-wt-2 and 1R-mt-wt-2) are compared to the distance pair descriptions from dataset groups (2R-wt-wt-1 and 2R-mt-wt-1) containing the local average information on equilibrium lengths, and the dataset groups (2R-wt-wt-2 and 2R-mt-wt-2) where average structural information is removed by shifting. Figure 3 shows typical examples of all three quantities plotted on the same graph for the *JS* and *KLave* measures respectively in panel 3a and 3b. Clearly the shifting procedure is needed to more accurately reflect protein dynamics. After the shifting is performed, the residue-residue distance pair information looks very similar to the on-residue measure. This leads to the question of whether the two types of descriptions (2R versus 1R) are redundant, meaning they linearly track each other. In particular, Figure 3c,d panels show scatter plots of 2R descriptions on the *y*-axis and 1R descriptions on the *x*-axis.

The large amount of scatter observed indicates 2R descriptions have orthogonal information content to the 1R descriptions. Nevertheless, the shifted data has somewhat less scatter and, more importantly, a linear trend line appears to be present within the midst of this scatter. This suggests quantifying protein dynamics in terms of fluctuations within distance pairs will yield similar, yet non-redundant, information as the on-residue description, and therefore its utility is likely to differ.

### 3.5. Kullback-Leibler Measure by Residue

**Figure 4:**
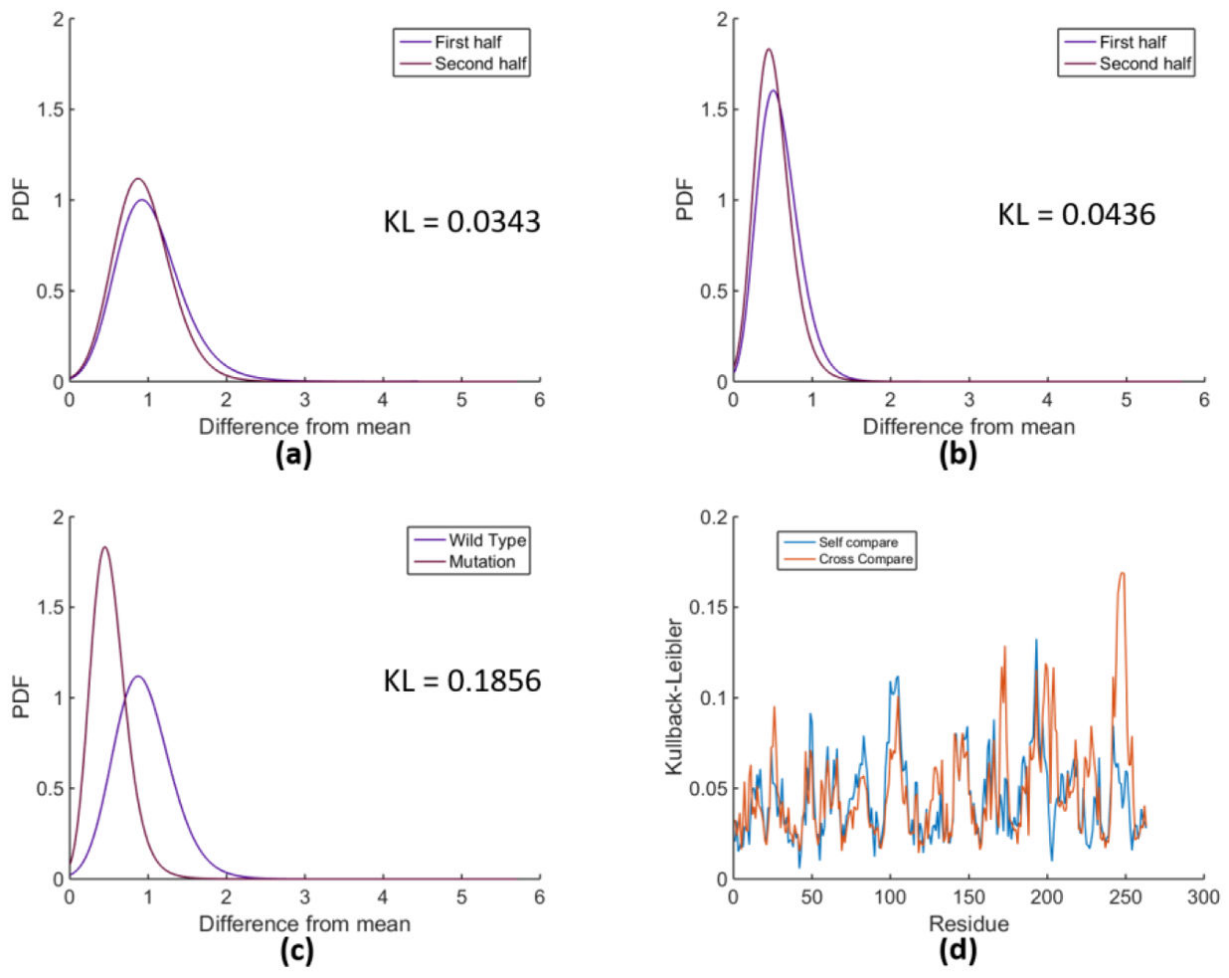
Comparison between residue 248 of wt-1erm and mt-1erm using KLave measure. (**a**) PDFs for residue 248 in the first and second half of wt-1erm; (**b**) PDFs for residue 248 in the first and second half of mt-1erm; (**c**) PDFs for residue 248 in the first half of the wt-1erm compared to the first half of mt-1erm; (**d**) KLave comparing averages of 2 self-comparisons and 4 cross-comparisons for all residues.

RMSF calculations are frequently used to compare two different MD trajectories by plotting the deviations per residue. Similar plots can be analyzed using any of the metrics we have defined to compare probability distributions. By calculating the distributions, however, we have the ability to visualize how the distributions differ for each residue. Presumably different metrics quantitatively capture differences in these distributions, but as seen above many do about as good as others, although certain metrics emphasize certain aspects more strongly than others. Figure 4 shows an example comparison between wt-1erm and mt-1erm trajectories, in which residue 248 has a notably different distribution between the wildtype and the mutation, compared to the fluctuations between the first and second halves within each trajectory.

### 3.6. Statistical Significance Descriptions

Since we calculate the *KS*1 measure in both the 1R and 2R descriptions, it is straightforward to obtain the corresponding *p*-values. The extraordinarily low *p*-values indicate that each MD trajectory, as we simulated them, are not similar at all. Specifically, the null hypothesis that the statistical properties of these local variables are the same between a pair of MD trajectories is rejected for almost all residues. This indicates wt to wt comparisons as well as self-convergence comparisons (data not shown) have essentially no statistical significance. However, we decided to plot log_10_(*pv*), where *pv* is the *p*-value, as a function of residue number for wt to wt comparisons and wt to mt comparisons using a 1R description. Additional results for these types of comparison plots are shown in supporting information, Figure S2.

**Figure 5:**
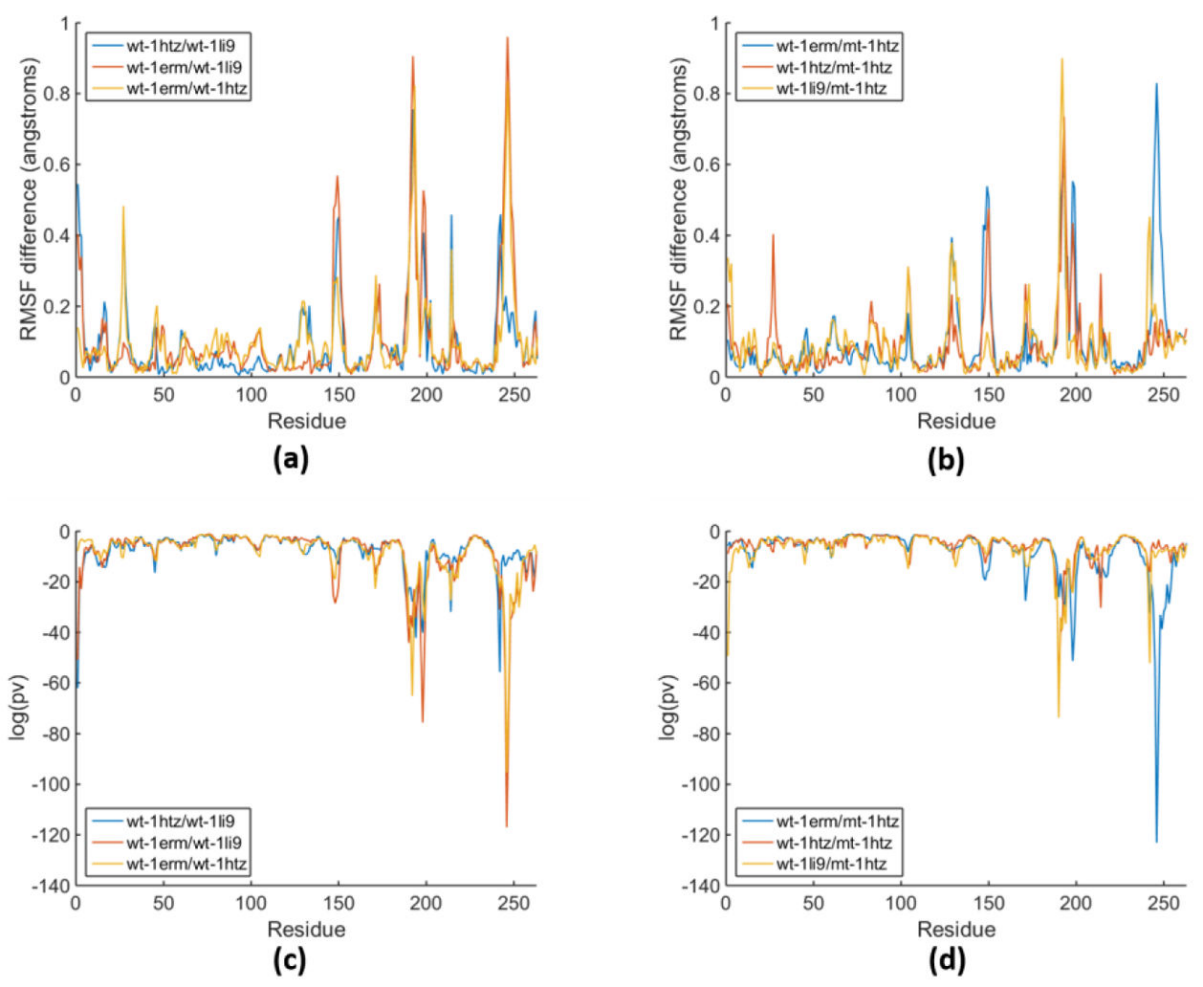
Comparison of RMSF differences and log_10_(*pv*) for local variables along the backbone of a protein. (**a**) RMSF difference for wt-wt comparisons difference; (**b**) RMSF difference for mutant with structure 1htz to wt comparisons (**c**) statistical significance of wt-wt comparisons; (**d**) statistical significance of mutant with structure 1htz to wt comparisons.

In Figure 5, RMSF and statistical significance are plotted together for two examples to show that qualitatively, both types of plots convey similar information. However, as one interprets these differences, it appears that differences in RMSF may have important functional consequences, while the statistical significance plots simply tells us that one MD trajectory is doing something very different compared to the other, at least within the local variables that are being compared. It may be that these two types of information complement each other (providing meaning why RMSF in the two MD trajectories are different) or perhaps, RMSF is different, but may have no consequence in its relationship to a functional mechanism. While qualitative similarity is found between the usual RMSF differences and the log_10_(*pv*) description along the protein backbone, it is also noted (c.f. Figure 1a) that these two quantities do not linearly correlate well, and convey somewhat different information.

**Figure 6:**
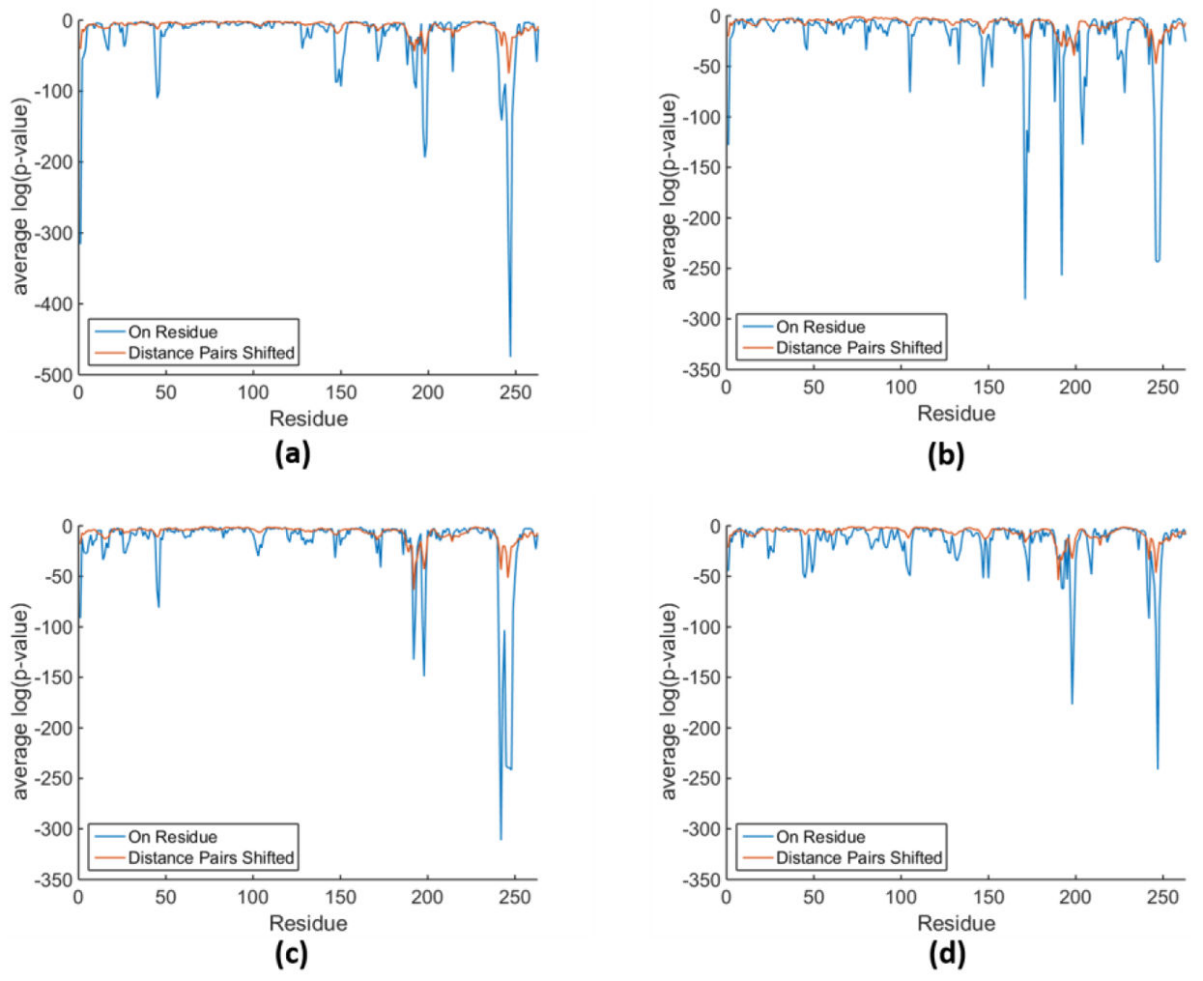
Statistical significance in terms of log_10_(*pv*) of local variables along the backbone of a protein comparing 1R and 2R descriptions. (**a**) wt-wt comparisons; (**b**) mutant with structure 1erm to wt comparisons; (**c**) mutant with structure 1htz to wt comparisons; (**d**) mutant with structure 1li9 to wt comparisons.

In Figure 6, we compare the statistical significance plots in terms of log_10_(*pv*) for the same cases we showed in Figure S2 except that the results are averaged over the dataset groupings rather than plotting each curve separately. The question addressed here is how different are the results using the 1R and 2R descriptors? While there is some qualitative shadowing, it is seen that these two types of descriptions have statistical significance on very different scales. In particular, R1 produces much lower *p*-values, indicating extreme differences, whereas 2R minimizes these differences somewhat. In particular, the 1R description shows numerous sharp dips, whereas the 2R description in the same place along the backbone often is void of these dips, or the magnitude is greatly diminished. On the various panels in Figure 6, it appears that there can be large segments of the protein that have similar local dynamics. However, the associated *p*-values are lower than 0.05 almost always. Nevertheless, the log_10_(*pv*) analysis appears to provide a well-defined quantitative comparison of (dis-)similarity between two MD trajectories along the backbone of a protein.

## 4. Discussion

Several different metrics were considered to quantify differences/similarities between MD trajectories, and overall, they produced markedly similar results. However, comparisons in terms of local variables at the residue level shows overwhelming statistical significance for sampling different distributions within different time windows. This result holds up between all groupings of datasets that includes comparing mutant to wildtype, wildtype to wildtype as a control, and self-comparisons involving first and second halves of the same MD trajectory. Nevertheless, the extraordinarily low *p*-values that were uncovered are surprising to us. We first discuss the correctness of these results, and then its relevance.

We first question the tactfully made assumption that the random variables are independently distributed. With a sampling rate of one frame per 50 ps we would expect the 1000 samples collected in each half of the trajectory are likely to retain some level of time-correlations that we ignore in our analysis. Therefore, is it possible that correlations are responsible for extremely low *p*-values? To check this, we created a test dataset in MATLAB using three Gaussian PDFs per residue that define the mean and variance in position for the *x*, *y*, and *z* directions. We simulated mock data using independent and identically distributed random variables for 263 “residues”. This test dataset has the exact structure as our dataset groupings. We find that shifting the mean and variance of the Gaussian distributions between corresponding residues of two “trajectories” always yields *p*-values that are vanishing low on the order of 10^−250^ to 10^−10^ depending on how much variance is in the mean and variance parameters across two test-”trajectories”. We note that with identical Gaussians, *p*-values uniformly spread on the interval [0, 1] as expected. However, for any deviation in Gaussian parameters that are consistent with uncertainties within the MD data, extraordinarily low *p*-values always appear. This motivated us to shuffle the time-ordered MD trajectory data and re-analyze it using identical procedure. Under uniform random shuffling the majority of *p*-values are greater than 0.05 when performing self-comparisons for convergence. Thus, statistically significant differences are present in samples for corresponding C*α* positions between a pair of MD trajectory time-segments over 50 ns, and correlations present within this span are not the cause of this effect.

We observe that RMSF differences between proteins track qualitatively well with all our other statistical measures, and the p-value analysis on a log_10_ scale tracks RMSF well. However, wildtype to wildtype comparisons exhibit the same level of RMSF deviations, and extremely low *p*-values, as that of the mutant to wildtype comparisons. This is an indication that the driving force that is causing these differences has little or nothing to do with the mutations, and that the effect of the mutations is hidden within the time windows simulated. We find that the descriptors that involve local average properties of distance pairs (with shifting to remove structural influences) had much higher *p*-values (c.f. Figure 6). This result suggests increasing spatial averaging leads to greater similarity between MD trajectories. Recall that the procedure to map distance pair information onto on-residue information along the backbone requires a local averaging over nearest neighbors. For the case of 12 Å cutoff the number of nearest neighbors on average is about 20. As shown in Figure S1, similar results are obtained for 8 and 10 Å cutoffs, but lower cutoffs exhibit insufficient local spatial averaging wherever the number of neighbors is low. A 4 Å cutoff does not yield robust results and 6 Å is marginal. The method becomes substantially more demanding computationally beyond 12 Å, while sensitivity to local effects begins to reduce, thereby becoming less meaningful.

Testing for similarity between two MD trajectories in terms of variables describing on-residue and residue-residue dynamics conveys insightful information. Calculating *p*-values per residue indicates if local dynamics has converged over the timescale of the simulation (shuffling the data proves that the method is capable of this). However, it is extraordinarily unlikely to be the case. We also looked at the root mean squared deviation (RMSD) of protein conformations relative to the initial frame to infer if the simulation converged (results shown in Figure S3). However, to claim MD simulation has converged is a risky proposition. To illustrate this, graduate students looking at RMSD plots for 100 ns identified anywhere from half to all the simulations as converged, possibly based on if the student is an optimist or pessimist when factoring in their time of graduation (pun intended). Of course, an objective measure is needed since a visual test over a short time period can lead to a biased view. From this perspective, Figures S3 and S4 include a case where the simulation time was extended to 500 ns. Interestingly, a mutant showed a jump in its RMSD at about 300 ns, despite the consensus opinion that the 100 ns simulation converged, but, not knowing the future.

Our results from MD simulations taken together with our mock data indicate MD simulation provides incomplete sampling for local residue level dynamics. We can understand the reason why by considering the simulation model for the mock data in more detail. Gaussian distributions assigned to each residue will have deviations in their parameterizations (i.e., mean and standard deviations) from one another. The deviations in these parameters are not interpreted as due to sampling error, but rather because different conformations will experience a different environment, which affects dynamical responses throughout the protein. Shorter time periods of sampling will reduce deviations within the window of time, but then it would be even less likely to explore similar environments within these windows of time if two windows are separated by times much longer than the sampling window time. Alternatively, data can be collected over longer times, but then, the protein has more time to explore different environments, which will increase the range in parameter variance. Both examples demonstrate the difficulty in building consistency in statistical properties between two time-segments. For convergence to take place, consistency must emerge.

The best situation, although computationally expensive or prohibitive, would be to increase the sampling rate and run simulations for much longer times to obtain greater accuracy. In future work, we suggest *p*-values could be calculated for much longer time periods (using 50 ps between frames). However, based on mock simulation data, the *p*-values are likely to increase only up to a point, but they will remain extraordinarily low if environmental differences are the cause of the deviations in the local PDFs. As such, similarity is unlikely to be found until the timescale is sufficiently long for rare large-scale structural rearrangements to be fully captured many thousands of times. For example, rather than a single jump in RMSD (see Figure S4), there should be many thousands of such jumps up and down, such that the distribution for the waiting time between jumps can be obtained from sampling. Furthermore, at temperatures near the melting temperature, this implies that unfolding and folding events must be captured within a simulation as a typical fluctuation. Only in this case can the MD simulation explore the conformational ensemble corresponding to thermodynamic equilibrium, consistently equivalent to shuffling of the data.

With these results in perspective, it is not clear that essential dynamics will tell us anything meaningful about the effect of mutations if functional mechanisms occur on much longer timescales than the simulation time. We give a simple analogy to clarify the pitfall that occurs when comparing MD trajectories on relatively short timescales to infer how functional mechanisms might change due to mutation, or other types of perturbation on a protein. If an observer would like to capture the essence of an entire culture, they could go to a large shopping mall and observe how people act. The observer can infer certain broad aspects of the culture of the society, but they cannot eliminate the small sample size problem. For instance, is there enough diversity in the observed environments (different shops, restaurants, and so forth) to adequately see what people experience during their daily routines outside of the mall? Is there biasing of traits by the people living in that local region? Can the observed traits of people in one mall for a few hours be extrapolated to an entire population? The answer is obviously no, unless there is perfect uniformity across society and only if variation in environment is irrelevant (both highly unlikely).

The results from this work show that MD trajectories sample rare events. In particular, MD trajectories cover nearly zero measure of the total conformational space that is accessible to a protein over timescales that span many orders of magnitude. This result is consistent with the mounting evidence that equilibrium is never achieved over any relatively small sub-segment of time relative to the typical lifetime of a protein [56]. This unfortunate limitation placed on MD simulations applied to proteins is a truism. Of course, concerns about how to sample relevant parts of conformational space are not new. Our results highlight the importance of using next generation MD simulation methods that focus on covering more functionally relevant conformational space, and questions the common practice of inferring functional mechanisms based on RMSF differences alone. Going forward, the statistical significance in terms of log_10_ of *p*-values can be used to quantitatively benchmark relative similarity between MD trajectories.

## Supplementary Material

supplemental

## Figures and Tables

**Table 1:** Summary of grouped datasets to facilitate comparisons between proteins and measures.

Group	Alignment	Averaging	All Protein Comparisons within the Group
1R-self-1	self	local	A-A, B-B, C-C, X-X, Y-Y, Z-Z
1R-self-2 1	self	global	A-A, B-B, C-C, X-X, Y-Y, Z-Z
1R-wt-wt-1	self	local	A-B, A-C, B-C
1R-wt-wt-2	mean	local	A-B, A-C, B-C
1R-mt-wt-1	self	local	X-A, X-B, X-C, Y-A, Y-B, Y-C, Z-A, Z-B, Z-C
1R-mt-wt-2	mean	local	X-A, X-B, X-C, Y-A, Y-B, Y-C, Z-A, Z-B, Z-C
2R-wt-wt-1	N/A	local	A-B, A-C, B-C
2R-mt-wt-1	N/A	local	X-A, X-B, X-C, Y-A, Y-B, Y-C, Z-A, Z-B, Z-C
2R-wt-wt-2	N/A	shifting	A-B, A-C, B-C
2R-mt-wt-2	N/A	shifting	X-A, X-B, X-C, Y-A, Y-B, Y-C, Z-A, Z-B, Z-C

1 Data from this group is not shown in any graph because results are nearly the same as 1R-self-1.
