# Supplementary material for: Statistical Measures to Quantify Similarity between Molecular Dynamics Simulation Trajectories
Source: Entropy (Basel). Author manuscript; Available in PMC 2018 Nov 27. (PMC6258182; doi:10.3390/e19120646)
Supplement: supplemental [file NIHMS971949-supplement-supplemental.pdf]

Statistical Measures to Quantify Similarity Between Molecular Dynamics Simulation Trajectories

Jenny Farmer [1], Fareeha Kanwal [1], Nikita Nikulsin [2], Matthew C. B. Tsilimigras [1], and Donald J. Jacobs [2,3,*]

[1] Department of Bioinformatics and Genomics, University of North Carolina at Charlotte, Charlotte, NC 28223, USA
[2] Department of Physics and Optical Science, University of North Carolina at Charlotte, Charlotte, NC 28223, USA
[3] Center for Biomedical Engineering and Science, University of North Carolina at Charlotte, Charlotte, NC 28223, USA

Supporting Information

Cutoff Distance Sensitivity

The decision concerning the cutoff distance between distance pair comparisons is somewhat arbitrary. Cutoffs less than five angstroms often consist of the two nearest neighbors only, causing the measurements to be too sensitive to single distances between a pair of residues, whereas increasing this distance provides a smoother average amongst mutliple neighbors. Including too many pairs, however, may not capture localized affects as well. Additionally, computational time increases substantially, approaching $O(N^2)$, as the cutoff value increases to very large values. Figure S1 shows how some of the measurements typically vary with cutoff value for the example case of the wt-mut comparison of 1erm. Qualitatively similar results are found for cutoffs of 8 or 10 angstroms.

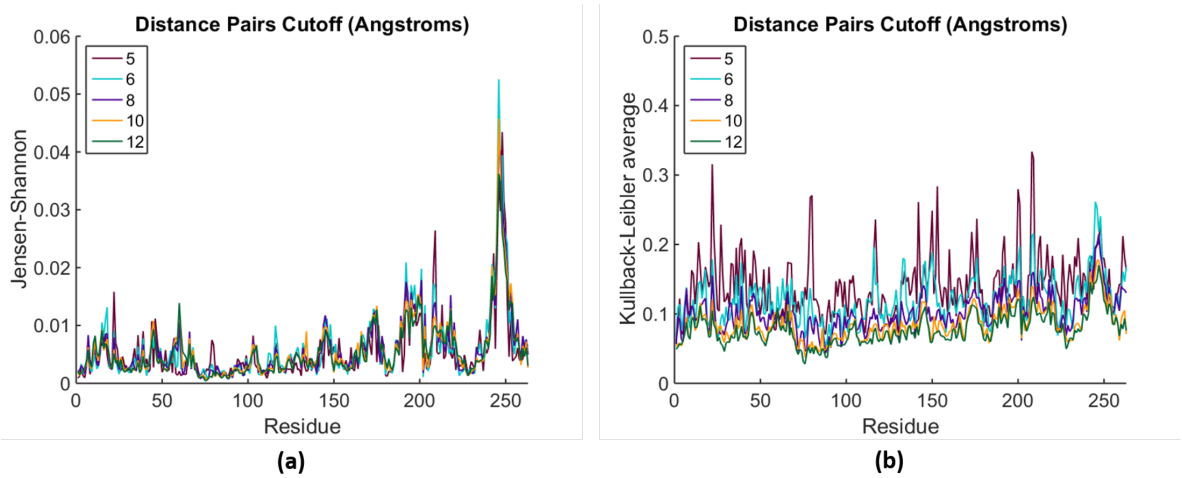

Figure S1. Comparisons between cutoff distances for distance-pairs for measures **(a)** *JS;* and **(b)** *KLave*

Statistical Significance For all Comparison Types

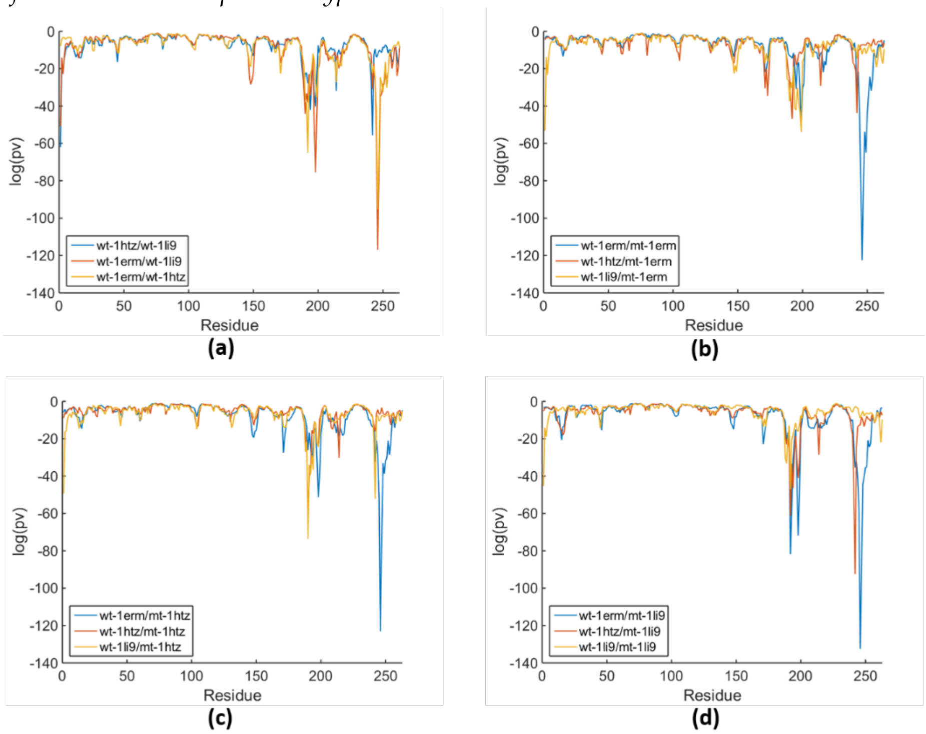

Figure S2. Statistical significance as $\log_{10}(pv)$ of local variables along the backbone of a protein. (**a**) wt-wt comparisons; (**b**) mutant with structure 1erm to wt comparisons; (**c**) mutant with structure 1htz to wt comparisons; (**d**) mutant with structure 1li9 to wt comparisons.

RMSD Comparisons and Convergence

The RMSD values and distributions for all six trajectories are shown in Figure S3. As convergence is inherantly difficult to confirm visually, an additional comparison was made for a much longer 500ns simulation of 1erm. Shown in purple are the last 2000 frames (last 100ns) of the 1erm trajectory. The RMSD values do not show obviously improved convergence even when the simulation is run for 5 times longer. Figure S4 further clarifies the difficulty of assessing convergence. The RMSD values for the entire 500ns simulation are shown for 1erm, comparing the wild type to the mutant. If only the first half of this simulation were available, it would probably be tempting to say that it seems to be converged, and yet the longer run time shows an abrupt change in RMSD for the mutant. The problem of convergence remains an unresolved concern in MD.

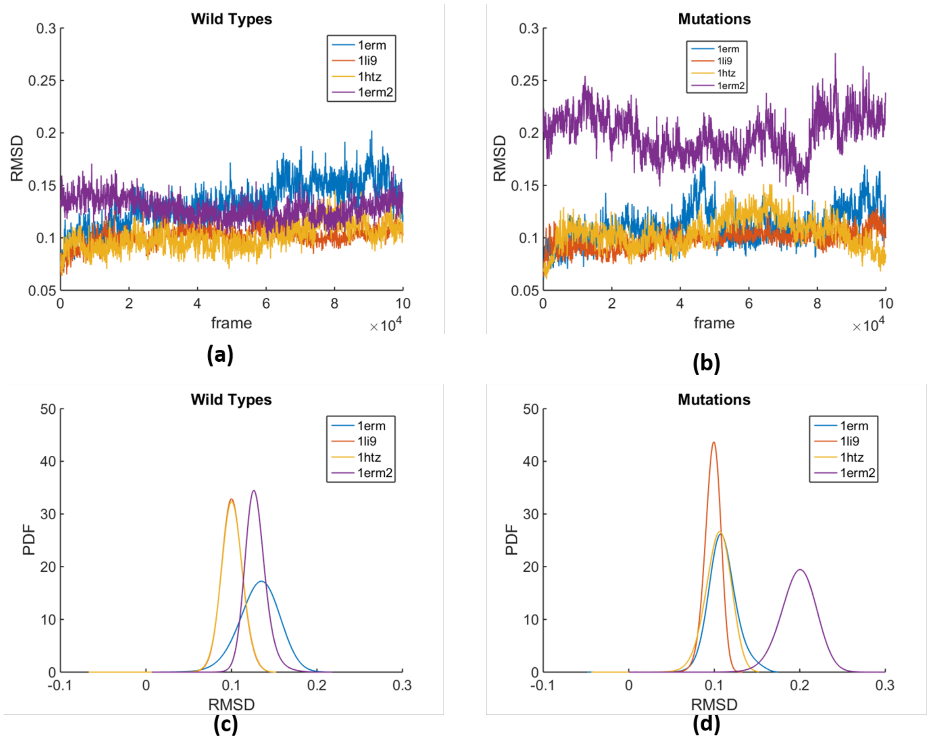

Figure S3. RMSD **(a)** as a function of time for wild type trajectories; **(b)** as a function of time for mutant trajectories; **(c)** distributions for wild type trajectories; **(d)** distributions for mutant trajectories.

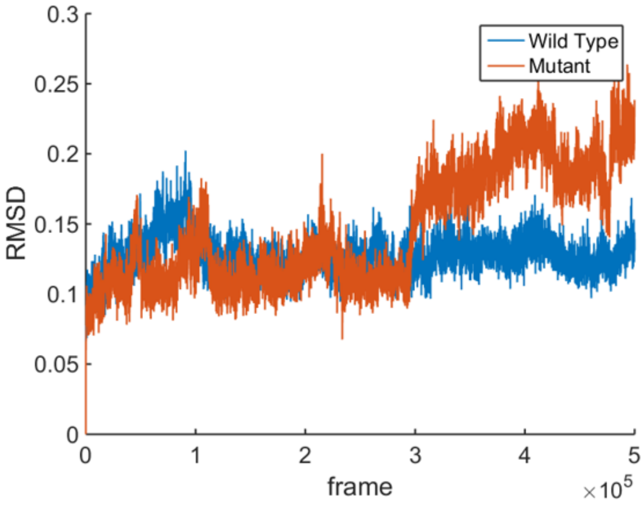

Figure S4. RMSD for 500ns trajectories comparing 1erm wild type and mutant
